# The Roles of AMPK in Revascularization

**DOI:** 10.1155/2020/4028635

**Published:** 2020-02-27

**Authors:** Ming-Hong Chen, Qiong-Mei Fu

**Affiliations:** ^1^Department of Geriatric Medicine, Xiangya Hospital, Central South University, Changsha 410008, China; ^2^Health Management Center, Xiangya Hospital, Central South University, Changsha 410008, China

## Abstract

Coronary heart disease (CHD) is the most common and serious illness in the world and has been researched for many years. However, there are still no real effective ways to prevent and save patients with this disease. When patients present with myocardial infarction, the most important step is to recover ischemic prefusion, which usually is accomplished by coronary artery bypass surgery, coronary artery intervention (PCI), or coronary artery bypass grafting (CABG). These are invasive procedures, and patients with extensive lesions cannot tolerate surgery. It is, therefore, extremely urgent to search for a noninvasive way to save ischemic myocardium. After suffering from ischemia, cardiac or skeletal muscle can partly recover blood flow through angiogenesis (de novo capillary) induced by hypoxia, arteriogenesis, or collateral growth (opening and remodeling of arterioles) triggered by dramatical increase of fluid shear stress (FSS). Evidence has shown that both of them are regulated by various crossed pathways, such as hypoxia-related pathways, cellular metabolism remodeling, inflammatory cells invasion and infiltration, or hemodynamical changes within the vascular wall, but still they do not find effective target for regulating revascularization at present. 5′-Adenosine monophosphate-activated protein kinase (AMPK), as a kinase, is not only an energy modulator but also a sensor of cellular oxygen-reduction substances, and many researches have suggested that AMPK plays an essential role in revascularization but the mechanism is not completely understood. Usually, AMPK can be activated by ADP or AMP, upstream kinases or other cytokines, and pharmacological agents, and then it phosphorylates key molecules that are involved in energy metabolism, autophagy, anti-inflammation, oxidative stress, and aging process to keep cellular homeostasis and finally keeps cell normal activity and function. This review makes a summary on the subunits, activation and downstream targets of AMPK, the mechanism of revascularization, the effects of AMPK in endothelial cells, angiogenesis, and arteriogenesis along with some prospects.

## 1. Introduction

Coronary heart disease (CHD) is the main cause of death globally; it is estimated that 17.9 million people died of cardiovascular diseases (CVDs) in 2016, representing 31% of all global deaths. The basic pathophysiology process is atherosclerosis, which tends to create plaque and block vascular cavity, resulting in myocardial ischemia, hypoxia or necrosis. Presently, the therapies for CHD mainly include coronary artery intervention (PCI) or coronary artery bypass grafting (CABG) [1]. However, postsurgical restenosis and low operative tolerance of aging and patients with extensive lesions limit its efficacy in CHD. Therefore, it is important to search for other alternative methods. Ischemic zones can actually recover blood perfusion by recruiting new vessels or expanding and remodeling produce arterioles; this process is also called revascularization and includes angiogenesis and arteriogenesis [2]. The mechanism of these processes has been widely studied. Angiogenesis is induced by hypoxia and involves three cells: tip cells, stalk cells, and phalanx cells [3–5], while the main stimulus of arteriogenesis is fluid shear stress (FSS), which is sensed by endothelial cells and consequently attracts leukocytes and promotes the phenotype transformation of vascular smooth muscle cells (VSMCs) [6–9]. Signal pathways of these two ways both include vascular endothelial growth factor (VEGF) pathway and nitric oxide- (NO-) dependent pathway [10–14] and both of them can be regulated by a highly conserved eukaryotic kinase, 5′adenosine monophosphate-activated protein kinase (AMPK) [15–17]; SNF1 and SnRK1 are its orthologues in yeast and several plants [18].

AMPK, a heterotrimeric complex combined by *α*, *β*, *γ* subunits, is activated by upstream kinases and regulated by the ratio of ADP/ATP or AMP/ATP or posttranslational modifications including phosphorylation and ubiquitylation, which exerts vital roles in maintaining energy homeostasis, protecting endothelial cellular function, regulating cellular autophagy, oxidative stress, and aging [19]. AMPK is ubiquitously expressed in a lot of tissues and cells, such as the endothelial cells (ECs), skeletal muscle, liver, and brain [20]. The roles of AMPK in revascularization have been widely researched, and it seems that the findings are varying in different conditions. In ischemia or hypoxia, AMPK activation facilitates angiogenesis but in tumor microenvironment inhibits it. Similarly, some findings show that AMPK promotes arteriogenesis by regulating inflammation but others suggest AMPK play a negative role in collateral circulation [15, 17].

## 2. AMPK

### 2.1. Subunits of AMPK

AMPK, a heterotrimeric protein complex, includes *α* subunit (encoded by protein kinase AMP-activated-*α* (PRKAA)) [21], *β* (PRKAB) [22], and *γ* (PRKAG) [23]. These isoforms play distinct roles in the AMPK stability and activity, but all three are essential for full activity. *α* (two isoforms) are catalytic subunits; *β* subunit (two isoforms) and *γ* subunit (three isoforms) contain the regulatory site, which could be combined by 12 various ways [24].

**Figure 1:**
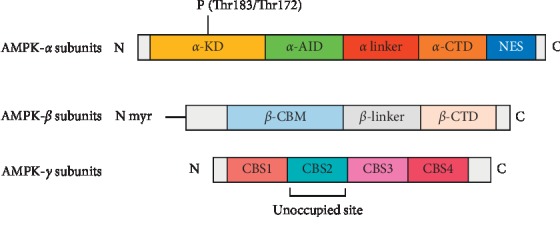
The structure of AMPK subunits: AMPK have three subunits, including *α*, *β*, *γ*. *α* is catalytic while *β* and *γ* are regulatory. Both *α*1 and *α*2 subunits have a crucial site in Thr183 and Thr172, whose phosphorylation is necessary for AMPK maximal activation. The *β* subunits could act as a scaffold, which makes the AMPK complex located on lysosomes, an exception from having phosphorylation, myristoylation, and carbohydrate-binding sites. The *γ* subunits bind the nucleotides by three sites, which are the structural basis for this energy sensor.

Both *α* subunits are similar in that their *N* termini have traditional serine/threonine kinase domains (*α*-KD) as well as the conserved threonine residue (*α*1 Thr183 and *α*2 Thr172), which are key phosphorylated sites [25]. The following are the inhibitory domains (*α*-AID), which negatively regulate AMPK. The C termini of AMPK is C-terminal domain (*α*-CTD) with nuclear export sequence (NES), whose crystal structure has not been resolved. Between *α*-AID and *α*-CTD is “*α* linker,” which is locked around the *γ* subunit (Figure 1). These two isoforms have various subcellular locational pattern; *α*1 isoform majorly appears to distribute in the cytoplasm or to associate with the plasma membrane of carotid body type 1 cells. However, *α*2 prefers locating in the nuclei of some cell types, such as skeletal muscle [26]. They have specificity of tissue distribution; for instance, AMPK*α*1 isoform is in the adipose tissue [27] while skeletal muscle expresses much higher AMPK*α*2 [28]. Interestingly, ECs have both of these isoforms, although AMPK*α*1 predominates at a much higher level than AMPK*α*2 [29].

Most of the parts of *β* subunits are highly conserved except the first 65 residues of NH2-terminus. AMPK*β*1 is nearly expressed in all cell types while *β*2 is mainly distributed in muscle. From N-terminus to C-terminus, *β* subunits have myristoylated N-terminal regions, carbohydrate-binding modules (*β*-CBM), *β*-linker regions, and the C-terminal domains (*β*-CTD) (Figure 1) [22, 30]. The crystal structures of *β*-CBM and *β*-CTD are completely resolved but the structures of N-terminal regions and *β*-linker are still unclear. Significantly, there is compelling evidence that N-terminal myristoylation of *β* subunits plays an indispensable role in AMPK lysosomal localization and activation in an AMP/ADP/ATP-independent manner in the process of glucose depletion [31, 32]. And N-myristoylation of AMPK *β* subunits also controls T cell inflammatory function [33, 34]. Hardie et al. have demonstrated that glycogen inhibits AMPK activation by binding the *β*-CBM of AMPK, which suggest that AMPK equilibrates cellular energy by sensing not only the change of AMP/ATP or ADP/ATP but also glycogen [35]. *β*-CTD interacts with *γ* N-terminal regions, which let AMPK become an intact complex to exert its normal function [36].

Although *γ* subunits have different lengths (*γ*1 331 < *γ*3 489 < *γ*2 569 residues), each one shares the same COOH-terminal having about 300 residues, a variable N-terminal domain that interacts with *β*-CTD and four tandem repeats of a motif termed CBS repeat (Figure 1) [18]. Excepting CBS2 which is an unoccupied site, CBS1, CBS3, and CBS4 could be bound by AMP or ATP by different affinities, CBS1 site binds ATP with higher affinity, but CBS3 site has higher affinity for AMP, and CBS4 is believed to be a nonchangeable site; that is, it binds AMP irreversibly [37, 38]. Furthermore, different isoforms of *γ* subunits also have distinct affinity with AMP, such as *γ*3 which is the least sensitive [39]. Like *α* and *β* subunits, *γ* subunits also have tissue distribution specificity; *γ*1 subunit is widely expressed in all tissues, whereas *γ*2 and *γ*3 isoforms are mainly abundant in skeletal muscle [40].

In conclusion, both *α*1 and *α*2 subunits have a crucial site in Thr183 and Thr172, whose phosphorylation is necessary for AMPK maximal activation. The *β* subunits could act as a scaffold, which makes AMPK complex locate on lysosomes, except for having phosphorylation, myristoylation, and carbohydrate-binding sites [18]. The *γ* subunits bind the nucleotides by three sites, which are structural basis for this energy sensor. Most importantly, the catalytic features of *α* subunit and regulatory activity of *β* and *γ* subunits are all integrant for AMPK correct and normal activation.

### 2.2. Activation of AMPK

AMPK is activated mainly by three complementary mechanisms: (1) allosteric activation [41–43]; (2) phosphorylation of *α*1 Thr183 or *α*2 Thr172 [25]; and (3) inhibiting dephosphorylation of Thr183 or Thr172 [44].

Mammalian AMPK is sensitive to the changes of AMP/ATP or ADP/ATP. Therefore, any cellular metabolic process that reduced ATP levels or increased AMP/ADP can activate AMPK, such as hypoxia, glucose decrease, mitochondrial oxidative stress, or metabolic inhibition of ATP synthesis [20, 45]. However, Lin and Hardie et al. found that AMPK can be activated through an additional AMP-/ADP-independent mechanism in response to glucose reduction both in vivo and in vitro [31]. They demonstrated that different compartmentalized pools of AMPK are activated through distinct ways, which depends on the extent of elevation of cellular AMP [46]. Low increases in AMP activate AMPK only via the AMP-independent, AXIN-based manner in lysosomes, which is regulated by fructose-1,6-bisphosphate (FBP) levels. When FBP decreases, adolase is released and then interacts with vacuolar-type H + -ATPase (V-ATPase), Ragulator, and AMPK-AXIN-LKB1 and finally becomes a complex and activates AMPK. Mild concentrations of AMP also enlarge this to activate cytosolic AMPK by an AXIN-dependent pathway. By comparison, severe glucose starvation activates all pools of AMPK in the AMP-/ADP-dependent manner rather than AXIN. Researches demonstrated a space-time basis for hierarchical activation of AMPK in various compartments in the process of differing the extents of energy stress [47]. But the question of how the FBP-free status of adolase binds vacuolar-type H + -ATPase (V-ATPase) has not been illuminated. Excitedly, Lin and Hardie et al. recently suggested that transient receptor potential cation channels (TRPVs), in low glucose, relay the adolase to the reconfiguration of v-ATPase, activating AMPK [48]. Although *α* subunit is catalytic, more and more evidence finds that regulatory *β* and *γ* subunits also are essential for AMPK optimum function. For example, N-myristoylation of *β* subunits is necessary for lysosome location of AMPK complex [31].

Besides allosteric activation, upstream two major AMPK kinases, which are liver kinase B1 (LKB1) [48], also known as serine/threonine kinase 11 (STK11) or renal carcinoma antigen NY-REN-19, and the Ca^2+^/calmodulin-dependent protein kinase kinase *β* (CaMKK*β*) [49] can regulate AMPK*α* activity through a phosphorylated manner. Researches reveal phosphorylation of the *α* subunit can depend on, or independently of, its LKB1 activity. CaMKK*β* is activated by intracellular concentration of Ca^2+^ [50, 51]. Thus, stimuli that magnify this, such as bradykinin [52] and thrombin [53], also phosphorylate AMPK*α* subunit in an AMP-/ADP-independent way owing to increased CaMKK*β* activity. It is worth mentioning that ubiquitination modification also regulates AMPK*α* activation. Zhenkun Lou et al. have found that AMPK*α*1 or AMPK*α*2 ubiquitination blocks its phosphorylation by LKB1, which could be rescued by the deubiquitinase ubiquitin specific peptidase 10 (USP10) [54]. Other researchers also have shown that AMPK*α*2 is ubiquitinated by ubiquitin-conjugating enzyme E2O (UBE2O) in a mouse model of breast cancer, which activates the mammalian target of rapamycin-hypoxia inducible factor 1-*α* (mTOR-HIF1-*α*) pathway and triggers cancer growth [55]. Similarly, AMPK*α*1 is also ubiquitinated and degraded by MAGE-A3/6-TRIM28 E3 ubiquitin ligase complex [56].

Briefly, in the case of replete energy, that is, low AMP/ATP or ADP/ATP, phosphatases can keep AMPK*α*1 Thr183 or *α*2 Thr172 in an unphosphorylated state by accessing to it. However, when energy decreases, CBS of the AMPK *γ* subunit is occupied by AMP or ADP, which prohibits the phosphatases from dephosphorylating Thr183 or Thr172, therefore increasing AMPK activity. It is worth mentioning that unlike AMP, ADP has no conspicuous allosteric effect on AMPK [44, 57].

### 2.3. Downstream Targets of AMPK

Downstream targets of AMPK mainly include molecules involving glucose, lipid, protein metabolism or inflammation, oxidative stress, and aging process.

During lipid metabolism, once being activated, AMPK as a serine/threonine kinase phosphorylates some crucial molecules that regulate lipid metabolism, such as acetyl-CoA carboxylase (ACC) [58], 3-hydroxy-3-methyl-glutaryl-coenzyme A reductase (HMG-CoA reductase) [42], and sterol regulatory element-binding protein 1c (SREBP1c) [59]. Except for the above-mentioned molecules, evidence has shown that AMPK reduces hepatic steatosis in high-fat, high-sucrose (HFHS) diet-fed mice by interacting with and mediates phosphorylation of insulin-induced gene (Insig), a novel effector of AMPK, which plays a critical role in regulating intracellular cholesterol equilibrium [60]. Furthermore, activated AMPK also stimulates skeletal muscle to uptake glucose by phosphorylating Rab-GTPase-activating protein TBC1 domain family member 4 (TBC1D4), which ultimately induces fusion of glucose transporter type 4 (GLUT-4) vesicles with the plasma membrane [61], and phosphorylates 6-phosphofructo-2-kinase (PFK-2) [62], glycogen, and glycogen synthase to promote glycolysis and inhibit glycogen synthesis. In addition, AMPK suppresses the energy-intensive protein biosynthesis process by phosphorylating tuberous sclerosis complex 2 (TSC2) which regulates activity of mammalian target of rapamycin complex 1(mTORC1) promoting protein synthesis [20, 63]. AMPK regulates autophagy by directly and indirectly activating Unc-51 like autophagy activating kinase (ULK1) [64, 65] and mitochondrial biogenesis by regulating peroxisome proliferator-activated receptor gamma coactivator 1-alpha (PGC-1*α*) which in turn promotes gene transcription in the mitochondria [66, 67]. AMPK participates in the cellular redox regulation and anti-inflammation response. Hong Li et al. have depicted that the Cys130 and Cys174 of AMPK*α* is oxidized during energy stress, which could be inhibited by Thioredoxin1 (Trx1) and protects AMPK activation in ischemia [68, 69]. In some inflammatory disease, AMPK also impacts a positive role, such as allergic diseases [68], monosodium urate (MSU) crystal-induced inflammation [70], and synovitis [33]. The process of aging, involving inflammation, oxidative stress, metabolic disorder, and decrease of autophagic clearance, is of course using AMPK as a supervisor that orchestras all the pathways in order to resist bad effects of senescence [71]. For instance, skeletal muscle AMPK knockdown-aged mice show hypoglycemia and hyperketosis during fasting [72].

## 3. The Mechanism of Revascularization

After the initiation of ischemia, cardiac or skeletal muscle undergoes a series of molecules and hemodynamical changes triggered by hypoxia-related pathways [10], invasion and infiltration inflammatory cells [73, 74], and cellular metabolism remodeling [75, 76], to promote capillary neogenesis (angiogenesis), or arterioles remodeling (arteriogenesis or collateral circulation), and then eventually to restore blood perfusion of ischemic zones.

Angiogenesis is induced by hypoxia via HIF1-*α*, which depicts the formation of new capillaries by sprouting or splitting from preexistent vessels, which is different from vasculogenesis [3, 5]. The latter is a process of endothelial cells from mesoderm cell precursors which form primitive tubules during the embryonic phase [10, 77, 78]. The process of angiogenesis is completed mainly by three EC subtypes. (1) Firstly, “tip cells” featured migratory capability sense proangiogenic stimuli, such as VEGF, fibroblast growth factor (FGF), and led the newly forming vessel to sprout towards the source of the proangiogenic stimuli. (2) During the migration of the tip cells, proliferative “stalk cells” lengthen neovessels. When neighbouring vessels' sprouts meet and their tip cells fuse, an interconnected, closed, and functional lumen allowing blood flow is formed. (3) Next, the quiescent “phalanx cells” mature neovessels featured by a typical cobblestone shape. (4) Finally, in order to form a tighter vessel for proper stability and barrier function, pericytes secrete platelet-derived growth factor-B (PDGF-B) and subsequently recruit VSMC expressing PDGF receptor *β* [79, 80]. Recently, the roles of metabolism remodeling of endothelial cells in angiogenesis are attached by many researchers. For example, Katrien and Yiming Xu et al. have found that endothelial 6-phosphofructo-2-kinase/fructose-2,6-bisphosphatase, isoform 3, (PFKFB3) plays a critical role in vessel sprouting and angiogenesis [81, 82].

Arteriogenesis or collateral growth, being different from angiogenesis, is a process that the existing interconnected vascular branches between adjacent blood vessels expand and remodel triggered by FSS, which is induced by increased flow across the collateral bed; when the main coronary artery is occluded, the downstream pressure decreases, resulting in an increased pressure drop and flow velocity across collaterals [83–85]. The basic pathophysiological courses of arteriogenesis contain the following. (1) Endothelial cells sense elevated FSS, which is the initiated step of arteriogenesis formation, by some molecules including Trpv4 [86], actin-binding rho activating protein (Abra) [87], and then change morphology and express multiple genes mainly participating in attracting circulatory blood cells and promoting cells adhesion, such as selectins, chemokine (C-C motif) ligand 2 (CCL2), intercellular adhesion molecules (ICAM), vascular cell adhesion molecules (VCAM-1), and VEGF. (2) The second one is inflammatory cell invasion and infiltration; for example, Florian P. Limbourg et al. suggest that endothelium matures macrophage and controls macrophage differentiation via Notch signaling, which in turn promotes arteriole growth [88], and neutrophils signal is enhanced at early ischemic phase [89]. (3) The third is VSMC proliferation, migration, and phenotypic transformation [6, 7]. Although a considerable number of researches using multifarious animal models have uncovered the signaling pathways of arteriogenesis involving the VEGF, PDGF, NO, and rho-pathway [87, 90], clinical trials are somehow disappointing [91].

## 4. AMPK in Endothelial Cells

ECs, mostly remaining quiescent throughout adult life, retain the capacity to rapidly form new blood vessels in response to injury or in pathological conditions such as hypoxia, ischemic, and hemodynamic changes. They then can respond with suitable regulatory and control processes to maintain cellular or systematic homeostasis. Such responses contain secretion of angiogenic factors promoting proliferation, migration of ECs, differentiation of endothelial progenitor cells (EPCs), or remodeling of endothelial metabolism.

It is widely believed that ECs prefer generating ATP through oxidative phosphorylation to produce more energy (the ratio of ATP yielded by oxidative phosphorylation and glycolysis is 30 : 2 or 32 : 2). In fact, ECs have a lower mitochondrial content and depend primarily on glycolysis [92]. Although the level of ATP per glucose generated is relatively low, high glycolytic flux can generate more ATP at a faster rate than oxidative phosphorylation when glucose is sufficient and is positioned to shunt glucose into glycolysis side branches to synthesize macromolecule such as the hexosamine and pentose phosphate. More advantages of aerobic glycolysis in ECs may include (1) generating less reactive oxygen species (ROS) by decreasing aerobic oxidation, (2) preserving maximal amounts of oxygen to supply perivascular cells, (3) making ECs adapt hypoxic environment they will grow into, and (4) producing lactate which is a proangiogenic signaling molecule [80, 93–95]. Except for glucose, another fuel source for ECs is fatty acids. Given the fact that it modestly contributes total ATPs in ECs, the exact role of fatty acids in ECs is elusive at present and needs more attention in the future. For example, Ulrike et al. show that fatty acid synthase knockdown (FASN^KD^) in ECs impedes vessel sprouting by reducing proliferation [76]. AMPK, as an energy and embolism gauge, can also phosphorylate key rate-limiting enzymes of the above-mentioned anabolism pathways in ECs, and as such the relationship between the AMPK and the ECs metabolism in angiogenesis still needs to be lucubrated.

For amino acid metabolism, arginine is most broadly studied for its conversion to citrulline and NO. The latter is the essential signaling molecule for endothelial function, which is synthesized by endothelial NO synthase (eNOS). eNOS expression and activity are carefully regulated by multiple interconnected mechanisms at the transcriptional (binding of transcription factors, DNA methylation), posttranscriptional (primary transcript modifications, mRNA stability, and nucleocytoplasmatic transport), and posttranslational levels (phosphorylation, fatty acid acylation, and protein-protein interactions) [96]. Modification of phosphorylation is vital for eNOS activity. In this moment, AMPK is the only kinase identified that can probably phosphorylate eNOS on more than one site, that is, Ser1177 and Ser633 in the reductase domain and inhibitory Thr495 site in the CaM-binding domain of the enzyme. A body of researches have reported AMPK dependent eNOS phosphorylation (on Ser1177) can proceed the following diverse endothelial cell stimulation, such as peroxisome proliferator-activated receptors (PPAR) agonists, AICAR, metformin, VEGF, and adiponectin. It is worth noting that the effects are usually weaker and much less arresting than other stimulation, like thrombin, hypoxia, and shear stress, which also lead to AMPK activation [97, 98].

## 5. AMPK in Angiogenesis

**Figure 2:**
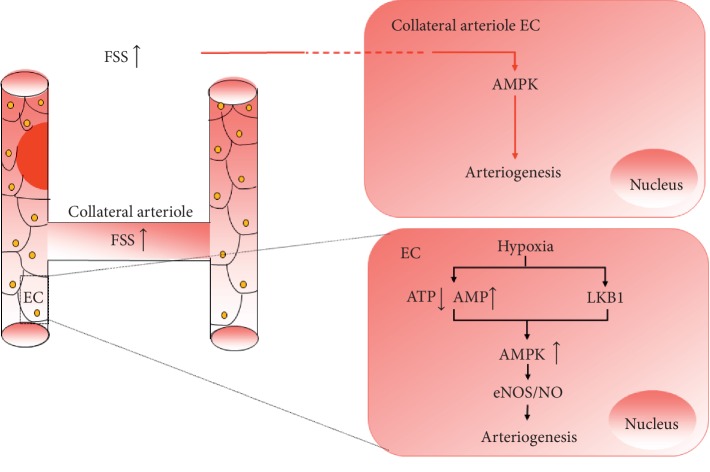
The roles of AMPK in revascularization. After vessels are occluded, remote tissues suffer ischemia and hypoxia, the blood perfusion of collateral arterioles increases, and the FSS is elevated. FSS and hypoxia activate AMPK by different or the same ways.

The roles of AMPK in angiogenesis have not been clarified and somehow are contradictory. A considerable amount of evidence has shown that AMPK exerts its positive impact on angiogenesis mainly in the metabolic syndrome, ischemia diseases, and hypoxia. That mainly includes four parts. (1) It guarantees energy supply of endothelial cells. (2) AMPK regulates EPCs differentiation, ECs proliferation, and migration [99, 100]. (3) AMPK, acting as an upstream kinase, phosphorylates eNOS to produce NO, facilitating vascular vasodilation and angiogenesis [101]. (4) Activation of AMPK under hypoxic conditions promotes autophagy, which somehow enhances VEGF expression [102]. Some earlier studies report that AMPK*α*1 impedes anoxia-induced apoptosis [103, 104] and protects against diabetes mellitus-induced vascular injury by improving EPCs function and promoting reendothelialization through upregulation of heme oxygenase-1 and stromal cell-derived factor 1 (SDF1) [105, 106], and dominant negative AMPK mutants inhibit both ECs migration and differentiation in vitro under hypoxia and in vivo angiogenesis [103]. In addition, evidence has demonstrated that LKB1/AMPK improve blood perfusion by inducing angiogenesis in hind limbs ischemic model of mice [102, 107] (Figure 2). At present, protective roles of AMPK in angiogenesis or on ECs or EPCs under some adverse condition, such as anoxia, stroke, senescence, and oxidative stress, have been validated [20, 108], and it also can be stimulated by cytokines or pharmacological agents such as VEGF [109], AICAR [109], metformin [100], berberine [110, 111], and adiponectin [112].

However, other researches have also revealed the passive effects of AMPK on angiogenesis. Evidence has demonstrated that AMPK exerts protective roles on retinopathy. Activated AMPK protects retinal vasculature from edema, hemorrhage, and final retinal detachment by decreasing oxidative stress and inflammation, improving circulation in narrow arterioles, inhibiting angiogenesis [113–116]. Studies have shown that metformin inhibits laser-induced choroidal neovascularization by activating AMPK [117]. Similarly, AMPK, being activated by berberine, can inhibit modified LDL-induced injury of Müller cell [118], which is the major glia of the retina; they are maintaining the blood-retinal barriers (BRBs). In addition, a variety of researches have shown that AMPK activation by many pharmacological activators, such as compound C, metformin, AICAR, curcumin, and simvastatin, inhibits tumor invasion and metastasis via the blockage of angiogenesis [119–122]. Furthermore, antifungal drug itraconazole targets mitochondrial protein voltage-dependent anion channel 1 (VDAC1) to suppress angiogenesis by modulating the AMPK/mTOR signaling axis in endothelial cells [123]. Interestingly, there are some studies which have shown that AMPK activation by some agents may play a positive role in tumor growth, even including metformin [124, 125].

Whether AMPK activation promotes angiogenesis or inhibits it depends on different cellular microenvironment. Generally, activation of AMPK in ischemic or hypoxic conditions facilitates angiogenesis but in tumor microenvironment inhibits it, which is attributed to different pathway activation. For example, under ischemic or hypoxic condition, AMPK activation has a positive effect on autophagy by inhibiting mTOR and phosphorylating autophagy modulators [126]. Autophagy somehow stabilizes HIF-1*α*, which regulates VEGF and other angiogenic molecules, and promotes angiogenesis [127]. The signal pathway of mTOR-HIF-1*α*-VEGF is activated in cancer cells; metformin or other AMPK activators can impede them, inhibiting angiogenesis [128].

## 6. AMPK in Arteriogenesis

So far, there is not much evidence on the role of AMPK in arteriogenesis and the ones that exist are inconsistent. One line of evidence shows that AMPK*α*1(–/–) can impair adult arteriogenesis in that it reduces accumulation of macrophages in ischemic hindlimb and inhibits the expression of growth factors in macrophages [15]. However, another has shown that mitochondrial oxidative stress impedes coronary collateral growth in lean rats in response to repetitive ischemia through activating AMPK and consequently inhibiting mTOR signaling, which is necessary for new protein synthesis and phenotypic switching of endothelial cells [17]. These two cases hint that the effects of AMPK in arteriogenesis under different physiological or pathological circumstances need to be developed further. Researches have shown that FSS, as a key factor which promotes opening and remodeling of collateral circulation, could influence activity of AMPK. For example, Wei Yi et al. have found that FSS can impede the survival and increase the apoptosis of bone marrow mesenchymal stem cells (BMSCs), which partly is attributed to the decrease of AMPK phosphorylation [129, 130]. What is more, exercise, also as an important element for arteriogenesis [131], has been found to play a positive role in AMPK activation. Young has verified that, in physiological condition, rat cardiac AMPK activity increases progressively with exercise intensity [132]. More importantly, Ferguson has also found that interval and continuous sprint cycling promotes phosphorylation of human skeletal muscle AMPK *α*Thr172 [133] (Figure 2).

## 7. Prospect

AMPK, as a key modulator of cellular energy, metabolism, and oxidative-redox homeostasis, plays a complicated regulatory role in the ECs. When AMPK is activated by elevated ratio of AMP/ATP or ADP/ATP, ROS, cytokines, or agents, the kinase will promote catalysis pathways, such as glycolysis, inhibit analysis pathways, such as glycogen or protein synthesis, and regulate inflammatory process and oxidative stress, through phosphorylation of some crucial enzymes such as eNOS, FASN, ACC, PFK-2, mTORC1, and ULK1. Although AMPK also participates in regulating revascularization, the effect of AMPK is contradictory. Generally, activated AMPK promotes angiogenesis in ischemia whereas inhibiting angiogenesis under retinopathy or tumor microenvironment. The role of AMPK during arteriogenesis also is double-faced, which is attributed to different intracellular or extracellular circumstances. Global knockout of AMPK*α*1 and macrophage-specific knockout mice, which are subjected to hindlimb ischemia brought about by femoral artery ligation, impairs adult arteriogenesis so that it reduces perfusion to the lower limb. However, if cells suffer mitochondrial oxidative stress, activated AMPK does not promote collateral growth; on the contrary, it suppresses arteriole opening or remodeling. As mentioned previously, although up until this moment there is no enough evidence that has shown the definite role of AMPK in arteriogenesis; given that both FSS and exercise also regulate AMPK phosphorylation, it is still worthy of exploring AMPK function in collateral circulation. What is more, AMPK, as a heterotrimeric protein complex, so far, has had many studies focus on the function of AMPK phosphorylation, while the role of other posttranslational modifications in revascularization need to be illuminated, such as ubiquitination, acetylization, and glycosylation. Different isoforms of AMPK may influence this process.

## Abbreviations

Abra: Actin-binding rho activating protein
ACC: Phosphorylates acetyl-CoA carboxylase
AMPK: 5′-Adenosine monophosphate-activated protein kinase
BMSCs: Bone marrow mesenchymal stem cells
BRBs: Blood-retinal barriers
CABG: Coronary artery bypass grafting
CaMKK*β*: Ca^2+^/calmodulin-dependent protein kinase kinase *β*
CCL2: Chemokine (C-C motif) ligand 2
CHD: Coronary heart disease
CVDs: Cardiovascular diseases
eNOS: Endothelial NO synthase
ECs: Endothelial cells
EPCs: Endothelial progenitor cells
FASN: Fatty acid synthase
FBP: Fructose 1,6-bisphosphate (FBP)
FBPase-2: 6-Phosphofructo-2-kinase
FGF: Fibroblast growth factor
FSS: Fluid shear stress
GLUT-4: Glucose transporter type 4
HIF1-*α*: Hypoxia inducible factor 1-*α*
HMG-CoA reductase: 3-Hydroxy-3-methyl-glutaryl-coenzyme A reductase
HNF4: Hepatocyte nuclear factor 4
ICAM: Intercellular adhesion molecules
Insig: Insulin-induced gene
LKB1: Liver kinase B1
MSU: Monosodium urate
mTORC1: Rapamycin complex 1
NO: Nitric oxide
PCI: Coronary artery intervention
PDGF: Platelet-derived growth factor
PFKFB3: Fructose-2,6-bisphosphatase, isoform 3
PGC-1*α*: Peroxisome proliferator-activated receptor gamma coactivator 1-alpha
PPAR: Peroxisome proliferator-activated receptors
ROS: Reactive oxygen species
SDF1: Stromal cell-derived factor 1
SREBP1c: Sterol regulatory element-binding protein 1c
STK11: Serine/threonine kinase 11
TBC1D4: TBC1 domain family member 4
TRPVs: Transient receptor potential cation channels
Trx1: Thioredoxin1
TSC2: Tuberous sclerosis complex 2
UBE2O: Ubiquitin-conjugating enzyme E2O
ULK1: Unc-51 like autophagy activating kinase
USP10: deubiquitinase ubiquitin specific peptidase 10
V-ATPase: Vacuolar-type H + -ATPase
VCAM-1: Vascular cell adhesion molecules
VDAC1: Voltage-dependent anion channel 1
VEGF: Vascular endothelial growth factor
VSMCs: Vascular smooth muscle cells.

## References

[1] Sipahi I., Akay M. H., Dagdelen S., Blitz A., Alhan C. (2014). Coronary artery bypass grafting vs percutaneous coronary intervention and long-term mortality and morbidity in multivessel disease. *JAMA Internal Medicine*.

[2] Silvestre J.-S., Smadja D. M., Lévy B. I. (2013). Postischemic revascularization: from cellular and molecular mechanisms to clinical applications. *Physiological Reviews*.

[3] Eelen G., de Zeeuw P., Treps L., Harjes U., Wong B. W., Carmeliet P. (2018). Endothelial cell metabolism. *Physiological Reviews*.

[4] Draoui N., de Zeeuw P., Carmeliet P. (2017). Angiogenesis revisited from a metabolic perspective: role and therapeutic implications of endothelial cell metabolism. *Open Biology*.

[5] De Bock K., Georgiadou M., Carmeliet P. (2013). Role of endothelial cell metabolism in vessel sprouting. *Cell Metabolism*.

[6] Zimarino M., D’Andreamatteo M., Waksman R., Epstein S. E., De Caterina R. (2014). The dynamics of the coronary collateral circulation. *Nature Reviews Cardiology*.

[7] Cai W., Schaper W. (2008). Mechanisms of arteriogenesis. *Acta Biochimica et Biophysica Sinica*.

[8] Meier P., Schirmer S. H., Lansky A. J. (2013). The collateral circulation of the heart. *BMC Medicine*.

[9] Heil M., Schaper W. (2004). Influence of mechanical, cellular, and molecular factors on collateral artery growth (arteriogenesis). *Circulation Research*.

[10] Rizzi A., Benagiano V., Ribatti D. (2017). Angiogenesis versus arteriogenesis. *Romanian Journal of Morphology and Embryology*.

[11] Rattner A., Williams J., Nathans J. (2019). Roles of HIFs and VEGF in angiogenesis in the retina and brain. *The Journal of Clinical Investigation*.

[12] Morrison A. R., Yarovinsky T. O., Young B. D. (2014). Chemokine-coupled *β*2 integrin-induced macrophage Rac2-Myosin IIA interaction regulates VEGF-A mRNA stability and arteriogenesis. *The Journal of Experimental Medicine*.

[13] Dai X., Faber J. E. (2010). Endothelial nitric oxide synthase deficiency causes collateral vessel rarefaction and impairs activation of a cell cycle gene network during arteriogenesis. *Circulation Research*.

[14] Lautz T., Lasch M., Borgolte J. (2018). Midkine controls arteriogenesis by regulating the bioavailability of vascular endothelial growth factor A and the expression of nitric oxide synthase 1 and 3. *EBioMedicine*.

[15] Zhu H., Zhang M., Liu Z. (2016). AMP-activated protein kinase *α*1 in macrophages promotes collateral remodeling and arteriogenesis in mice in vivo. *Arteriosclerosis, Thrombosis, and Vascular Biology*.

[16] Li Y., Sun R., Zou J. (2019). Dual roles of the AMP-activated protein kinase pathway in angiogenesis. *Cells*.

[17] Pung Y. F., Sam W. J., Stevanov K. (2013). Mitochondrial oxidative stress corrupts coronary collateral growth by activating adenosine monophosphate activated kinase-*α* signaling. *Arteriosclerosis, Thrombosis, and Vascular Biology*.

[18] Lin S.-C., Hardie D. G. (2018). AMPK: sensing glucose as well as cellular energy status. *Cell Metabolism*.

[19] Hardie D. G., Lin S.-C. (2017). AMP-activated protein kinase-not just an energy sensor. *F1000Research*.

[20] Jeon S.-M. (2016). Regulation and function of AMPK in physiology and diseases. *Experimental & Molecular Medicine*.

[21] Stapleton D., Gao G., Michell B. J. (1994). Mammalian 5’-AMP-activated protein kinase non-catalytic subunits are homologs of proteins that interact with yeast Snf1 protein kinase. *Journal of Biological Chemistry*.

[22] Thornton C., Snowden M. A., Carling D. (1998). Identification of a novel AMP-activated protein kinase *β* subunit isoform that is highly expressed in skeletal muscle. *Journal of Biological Chemistry*.

[23] Cheung P. C. F., Salt I. P., Davies S. P., Hardie D. G., Carling D. (2000). Characterization of AMP-activated protein kinase *γ*-subunit isoforms and their role in AMP binding. *Biochemical Journal*.

[24] Myers R. W., Guan H.-P., Ehrhart J. (2017). Systemic pan-AMPK activator MK-8722 improves glucose homeostasis but induces cardiac hypertrophy. *Science*.

[25] Hawley S. A., Davison M., Woods A. (1996). Characterization of the AMP-activated protein kinase kinase from rat liver and identification of threonine 172 as the major site at which it phosphorylates AMP-activated protein kinase. *Journal of Biological Chemistry*.

[26] Shirwany N. A., Zou M.-H. (2010). AMPK in cardiovascular health and disease. *Acta Pharmacologica Sinica*.

[27] Wang S., Liang X., Yang Q. (2015). Resveratrol induces brown-like adipocyte formation in white fat through activation of AMP-activated protein kinase (AMPK) *α*1. *International Journal of Obesity*.

[28] Thomson D. M. (2018). The role of AMPK in the regulation of skeletal muscle size, hypertrophy, and regeneration. *International Journal of Molecular Sciences*.

[29] Dong Y., Zhang M., Liang B. (2010). Reduction of AMP-activated protein kinase *α*2 increases endoplasmic reticulum stress and atherosclerosis in vivo. *Circulation*.

[30] Oakhill J. S., Chen Z.-P., Scott J. W. (2010). -Subunit myristoylation is the gatekeeper for initiating metabolic stress sensing by AMP-activated protein kinase (AMPK). *Proceedings of the National Academy of Sciences*.

[31] Zhang C.-S., Hawley S. A., Zong Y. (2017). Fructose-1,6-bisphosphate and aldolase mediate glucose sensing by AMPK. *Nature*.

[32] Li M., Zhang C. S., Zong Y. (2019). Transient receptor potential V channels are essential for glucose sensing by aldolase and AMPK. *Cell Metabolism*.

[33] Wen Z., Jin K., Shen Y. (2019). Transient receptor potential V channels are essential for glucose sensing by aldolase and AMPK. *Cell Metabolism*.

[34] Finlay D. K. (2019). N-myristoylation of AMPK controls T cell inflammatory function. *Nature Immunology*.

[35] McBride A., Ghilagaber S., Nikolaev A., Hardie D. G. (2009). The glycogen-binding domain on the AMPK beta subunit allows the kinase to act as a glycogen sensor. *Cell Metabolism*.

[36] Bateman A. (1997). The structure of a domain common to archaebacteria and the homocystinuria disease protein. *Trends in Biochemical Sciences*.

[37] Kemp B. E., Oakhill J. S., Scott J. W. (2007). AMPK structure and regulation from three angles. *Structure*.

[38] Xiao B., Heath R., Saiu P. (2007). Structural basis for AMP binding to mammalian AMP-activated protein kinase. *Nature*.

[39] Scott J. W., Hawley S. A., Green K. A. (2004). CBS domains form energy-sensing modules whose binding of adenosine ligands is disrupted by disease mutations. *Journal of Clinical Investigation*.

[40] Pinter K., Grignani R. T., Watkins H. (2013). Localisation of AMPK gamma subunits in cardiac and skeletal muscles. *Journal of Muscle Research and Cell Motility*.

[41] Yeh L. A., Lee K. H., Kim K. H. (1980). Regulation of rat liver acetyl-CoA carboxylase. Regulation of phosphorylation and inactivation of acetyl-CoA carboxylase by the adenylate energy charge. *Journal of Biological Chemistry*.

[42] Carling D., Zammit V. A., Hardie D. G. (1987). A common bicyclic protein kinase cascade inactivates the regulatory enzymes of fatty acid and cholesterol biosynthesis. *FEBS Letters*.

[43] Gowans G. J., Hawley S. A., Ross F. A., Hardie D. G. (2013). AMP is a true physiological regulator of AMP-activated protein kinase by both allosteric activation and enhancing net phosphorylation. *Cell Metabolism*.

[44] Hardie B., Sanders M. J., Underwood E. (2011). Structure of mammalian AMPK and its regulation by ADP. *Nature*.

[45] Steinberg G. R., Carling D. (2019). AMP-activated protein kinase: the current landscape for drug development. *Nature Reviews Drug Discovery*.

[46] Zong Y., Zhang C.-S., Li M. (2019). Hierarchical activation of compartmentalized pools of AMPK depends on severity of nutrient or energy stress. *Cell Research*.

[47] Carling D. (2019). AMPK hierarchy: a matter of space and time. *Cell Research*.

[48] Hawley S. A., Boudeau J., Reid J. L. (2003). Complexes between the LKB1 tumor suppressor, STRAD alpha/beta and MO25 alpha/beta are upstream kinases in the AMP-activated protein kinase cascade. *Journal of Biology*.

[49] Hurley R. L., Anderson K. A., Franzone J. M., Kemp B. E., Means A. R., Witters L. A. (2005). The Ca^2+^/calmodulin-dependent protein kinase kinases are AMP-activated protein kinase kinases. *Journal of Biological Chemistry*.

[50] Li S., Lavagnino Z., Lemacon D. (2019). Ca^2+^-Stimulated AMPK-dependent phosphorylation of Exo1 protects stressed replication forks from aberrant resection. *Molecular Cell*.

[51] Simoneau A., Zou L. (2019). Calcium influx guards replication forks against exonuclease 1. *Molecular Cell*.

[52] Mount P. F., Lane N., Venkatesan S. (2008). Bradykinin stimulates endothelial cell fatty acid oxidation by CaMKK-dependent activation of AMPK. *Atherosclerosis*.

[53] Stahmann N., Woods A., Carling D., Heller R. (2006). Thrombin activates AMP-activated protein kinase in endothelial cells via a pathway involving Ca^2+^/calmodulin-dependent protein kinase kinase. *Molecular and Cellular Biology*.

[54] Heller M., Yang X., Qin B. (2016). Deubiquitination and activation of AMPK by USP10. *Molecular Cell*.

[55] Vila I. K., Yao Y., Kim G. (2017). A UBE2O-AMPK*α*2 axis that promotes tumor initiation and progression offers opportunities for therapy. *Cancer Cell*.

[56] Pineda C. T., Ramanathan S., Fon Tacer K. (2015). Degradation of AMPK by a cancer-specific ubiquitin ligase. *Cell*.

[57] Carling D., Clarke P. R., Zammit V. A. (1989). Purification and characterization of the AMP-activated protein kinase. copurification of acetyl-CoA carboxylase kinase and 3-hydroxy-3-methylglutaryl-CoA reductase kinase activities. *European Journal of Biochemistry*.

[58] Fullerton M. D., Galic S., Marcinko K. (2013). Single phosphorylation sites in Acc1 and Acc2 regulate lipid homeostasis and the insulin-sensitizing effects of metformin. *Nature Medicine*.

[59] Bertolio R., Napoletano F., Mano M. (2019). Sterol regulatory element binding protein 1 couples mechanical cues and lipid metabolism. *Nature Communications*.

[60] Han Y., Hu Z., Cui A. (2019). Post-translational regulation of lipogenesis via AMPK-dependent phosphorylation of insulin-induced gene. *Nature Communications*.

[61] Kjobsted R., Treebak J. T., Fentz J. (2015). Prior AICAR stimulation increases insulin sensitivity in mouse skeletal muscle in an AMPK-dependent manner. *Diabetes*.

[62] Marsin A.-S., Bertrand† L., Rider M. H. (2000). Phosphorylation and activation of heart PFK-2 by AMPK has a role in the stimulation of glycolysis during ischaemia. *Current Biology*.

[63] Howell J. J., Hellberg K., Turner M. (2017). Metformin inhibits hepatic mTORC1 signaling via dose-dependent mechanisms involving AMPK and the TSC complex. *Cell Metabolism*.

[64] Laker R. C., Drake J. C., Wilson R. J. (2017). Ampk phosphorylation of Ulk1 is required for targeting of mitochondria to lysosomes in exercise-induced mitophagy. *Nature Communications*.

[65] Dite T. A., Ling N. X. Y., Scott J. W. (2017). The autophagy initiator ULK1 sensitizes AMPK to allosteric drugs. *Nature Communications*.

[66] Koh J.-H., Hancock C. R., Terada S., Higashida K., Holloszy J. O., Han D.-H. (2017). PPAR*β* is essential for maintaining normal levels of PGC-1*α* and mitochondria and for the increase in muscle mitochondria induced by exercise. *Cell Metabolism*.

[67] Viscomi C., Bottani E., Civiletto G. (2011). In vivo correction of COX deficiency by activation of the AMPK/PGC-1*α* axis. *Cell Metabolism*.

[68] Shao D., Oka S.-i., Liu T. (2014). A redox-dependent mechanism for regulation of AMPK activation by Thioredoxin1 during energy starvation. *Cell Metabolism*.

[69] Hwang S.-L., Li X., Lu Y. (2013). AMP-activated protein kinase negatively regulates Fc*ε*RI-mediated mast cell signaling and anaphylaxis in mice. *Journal of Allergy and Clinical Immunology*.

[70] Wang Y., Viollet B., Terkeltaub R., Liu-Bryan R. (2016). AMP-activated protein kinase suppresses urate crystal-induced inflammation and transduces colchicine effects in macrophages. *Annals of the Rheumatic Diseases*.

[71] Liu-Bryan A., Kaarniranta K. (2012). AMP-activated protein kinase (AMPK) controls the aging process via an integrated signaling network. *Ageing Research Reviews*.

[72] Bujak A. L., Crane J. D., Lally J. S. (2015). AMPK activation of muscle autophagy prevents fasting-induced hypoglycemia and myopathy during aging. *Cell Metabolism*.

[73] Jaipersad A. S., Lip G. Y. H., Silverman S., Shantsila E. (2014). The role of monocytes in angiogenesis and atherosclerosis. *Journal of the American College of Cardiology*.

[74] Shantsila A., Pontecorvo L., Agresta A., Rosano G., Stabile E. (2012). Regulation of collateral blood vessel development by the innate and adaptive immune system. *Trends in Molecular Medicine*.

[75] Eelen G., Dubois C., Cantelmo A. R. (2018). Role of glutamine synthetase in angiogenesis beyond glutamine synthesis. *Nature*.

[76] Bruning U., Morales-Rodriguez F., Kalucka J. (2018). Impairment of angiogenesis by fatty acid synthase inhibition involves mTOR malonylation. *Cell Metabolism*.

[77] Patel-Hett S., D’Amore P. A. (2011). Signal transduction in vasculogenesis and developmental angiogenesis. *The International Journal of Developmental Biology*.

[78] Ferguson J. E., Kelley R. W., Patterson C. (2005). Mechanisms of endothelial differentiation in embryonic vasculogenesis. *Arteriosclerosis, Thrombosis, and Vascular Biology*.

[79] Bierhansl L., Conradi L.-C., Treps L., Dewerchin M., Carmeliet P. (2017). Central role of metabolism in endothelial cell function and vascular disease. *Physiology*.

[80] Rohlenova K., Veys K., Miranda-Santos I., De Bock K., Carmeliet P. (2018). Endothelial cell metabolism in health and disease. *Trends in Cell Biology*.

[81] Xu Y., An X., Guo X. (2014). Endothelial PFKFB3 plays a critical role in angiogenesis. *Arteriosclerosis, Thrombosis, and Vascular Biology*.

[82] De Bock K., Georgiadou M., Schoors S. (2013). Role of PFKFB3-driven glycolysis in vessel sprouting. *Cell*.

[83] Deindl E., Schaper W. (2005). The art of arteriogenesis. *Cell Biochemistry and Biophysics*.

[84] Pipp F., Boehm S., Cai W.-J. (2004). Elevated fluid shear stress enhances postocclusive collateral artery growth and gene expression in the pig hind limb. *Arteriosclerosis, Thrombosis, and Vascular Biology*.

[85] Mack P. J., Zhang Y., Chung S., Vickerman V., Kamm R. D., García-Cardeña G. (2009). Biomechanical regulation of endothelium-dependent events critical for adaptive remodeling. *Journal of Biological Chemistry*.

[86] Troidl C., Troidl K., Schierling W. (2009). Trpv4 induces collateral vessel growth during regeneration of the arterial circulation. *Journal of Cellular and Molecular Medicine*.

[87] Troidl K., Rüding I., Cai W.-J. (2009). Actin-binding rho activating protein (Abra) is essential for fluid shear stress-induced arteriogenesis. *Arteriosclerosis, Thrombosis, and Vascular Biology*.

[88] Krishnasamy K., Limbourg A., Kapanadze T. (2017). Blood vessel control of macrophage maturation promotes arteriogenesis in ischemia. *Nature Communications*.

[89] Behm C. Z., Kaufmann B. A., Carr C. (2008). Molecular imaging of endothelial vascular cell adhesion molecule-1 expression and inflammatory cell recruitment during vasculogenesis and ischemia-mediated arteriogenesis. *Circulation*.

[90] Schaper W., Scholz D. (2003). Factors regulating arteriogenesis. *Arteriosclerosis, Thrombosis, and Vascular Biology*.

[91] Kikuchi R., Nakamura K., MacLauchlan S. (2014). An antiangiogenic isoform of VEGF-A contributes to impaired vascularization in peripheral artery disease. *Nature Medicine*.

[92] Groschner L. N., Waldeck-Weiermair M., Malli R., Graier W. F. (2012). Endothelial mitochondria-less respiration, more integration. *Pflügers Archiv-European Journal of Physiology*.

[93] Graier J. R., Haaga R. (2013). Acidic lactate sequentially induced lymphogenesis, phlebogenesis, and arteriogenesis (ALPHA) hypothesis: lactate-triggered glycolytic vasculogenesis that occurs in normoxia or hypoxia and complements the traditional concept of hypoxia-based vasculogenesis. *Surgery*.

[94] Yang L., Gao L., Nickel T. (2017). Lactate promotes synthetic phenotype in vascular smooth muscle cells. *Circulation Research*.

[95] Ghesquière B., Wong B. W., Kuchnio A., Carmeliet P. (2014). Metabolism of stromal and immune cells in health and disease. *Nature*.

[96] Carmeliet S. C., Robb G. B., Marsden P. A. (2004). Endothelial nitric oxide synthase. *Arteriosclerosis, Thrombosis, and Vascular Biology*.

[97] Zippel N., Loot A. E., Stingl H. (2018). Endothelial AMP-activated kinase alpha1 phosphorylates eNOS on Thr495 and decreases endothelial NO formation. *International Journal of Molecular Sciences*.

[98] Fleming I. (2010). Molecular mechanisms underlying the activation of eNOS. *Pflügers Archiv-European Journal of Physiology*.

[99] Li X., Han Y., Pang W. (2008). AMP-activated protein kinase promotes the differentiation of endothelial progenitor cells. *Arteriosclerosis, Thrombosis, and Vascular Biology*.

[100] Niu C., Chen Z., Kim K. T. (2019). Metformin alleviates hyperglycemia-induced endothelial impairment by downregulating autophagy via the Hedgehog pathway. *Autophagy*.

[101] Takahashi N., Shibata R., Ouchi N., Sugimoto M., Murohara T., Komori K. (2015). Metformin stimulates ischemia-induced revascularization through an eNOS dependent pathway in the ischemic hindlimb mice model. *Journal of Vascular Surgery*.

[102] Ouchi N., Shibata R., Walsh K. (2005). AMP-activated protein kinase signaling stimulates VEGF expression and angiogenesis in skeletal muscle. *Circulation Research*.

[103] Nagata D., Mogi M., Walsh K. (2003). AMP-activated protein kinase (AMPK) signaling in endothelial cells is essential for angiogenesis in response to hypoxic stress. *Journal of Biological Chemistry*.

[104] Nagata D., Kiyosue A., Takahashi M. (2009). A new constitutively active mutant of AMP-activated protein kinase inhibits anoxia-induced apoptosis of vascular endothelial cell. *Hypertension Research*.

[105] Li F. Y. L., Lam K. S. L., Tse H.-F. (2012). Endothelium-selective activation of AMP-activated protein kinase prevents diabetes mellitus-induced impairment in vascular function and reendothelialization via induction of heme oxygenase-1 in mice. *Circulation*.

[106] Yu J. W., Deng Y. P., Han X. (2016). Metformin improves the angiogenic functions of endothelial progenitor cells via activating AMPK/eNOS pathway in diabetic mice. *Cardiovascular Diabetology*.

[107] Ohashi K., Ouchi N., Higuchi A., Shaw R. J., Walsh K. (2010). LKB1 deficiency in Tie2-Cre-expressing cells impairs ischemia-induced angiogenesis. *Journal of Biological Chemistry*.

[108] Li J., McCullough L. D. (2010). Effects of AMP-activated protein kinase in cerebral ischemia. *Journal of Cerebral Blood Flow & Metabolism*.

[109] Zibrova D., Vandermoere F., Göransson O. (2017). GFAT1 phosphorylation by AMPK promotes VEGF-induced angiogenesis. *Biochemical Journal*.

[110] Zhu J., Cao D., Guo C. (2019). Berberine facilitates angiogenesis against ischemic stroke through modulating microglial polarization via AMPK signaling. *Cellular and Molecular Neurobiology*.

[111] Zhu M.-L., Yin Y.-L., Ping S. (2017). Berberine promotes ischemia-induced angiogenesis in mice heart via upregulation of microRNA-29b. *Clinical and Experimental Hypertension*.

[112] Wang S., Miao J., Qu M., Yang G.-Y., Shen L. (2017). Adiponectin modulates the function of endothelial progenitor cells via AMPK/eNOS signaling pathway. *Biochemical and Biophysical Research Communications*.

[113] Xu L., Kong L., Wang J., Ash J. D. (2018). Stimulation of AMPK prevents degeneration of photoreceptors and the retinal pigment epithelium. *Proceedings of the National Academy of Sciences*.

[114] Ash M., Inman D. M. (2018). Reduced AMPK activation and increased HCAR activation drive anti-inflammatory response and neuroprotection in glaucoma. *Journal of Neuroinflammation*.

[115] Athanasiou D., Aguila M., Opefi C. A. (2017). Rescue of mutant rhodopsin traffic by metformin-induced AMPK activation accelerates photoreceptor degeneration. *Human Molecular Genetics*.

[116] Tomizawa A., Hattori Y., Inoue T., Hattori S., Kasai K. (2011). Fenofibrate suppresses microvascular inflammation and apoptosis through adenosine monophosphate-activated protein kinase activation. *Metabolism*.

[117] Ying Y., Ueta T., Jiang S. (2017). Metformin inhibits ALK1-mediated angiogenesis via activation of AMPK. *Oncotarget*.

[118] Fu D., Yu J. Y., Connell A. R. (2016). Beneficial effects of berberine on oxidized LDL-induced cytotoxicity to human retinal müller cells. *Investigative Opthalmology & Visual Science*.

[119] Dasgupta B., Chhipa R. R. (2016). Evolving lessons on the complex role of AMPK in normal physiology and cancer. *Trends in Pharmacological Sciences*.

[120] Lee Y. T., Lim S. H., Lee B. (2019). Compound C inhibits B16-F1 tumor growth in a Syngeneic Mouse Model via the blockage of cell cycle progression and angiogenesis. *Cancers*.

[121] Wang J.-C., Li X.-X., Sun X. (2018). Activation of AMPK by simvastatin inhibited breast tumor angiogenesis via impeding HIF-1*α*-induced pro-angiogenic factor. *Cancer Science*.

[122] Bianchi G., Ravera S., Traverso C. (2018). Curcumin induces a fatal energetic impairment in tumor cells in vitro and in vivo by inhibiting ATP-synthase activity. *Carcinogenesis*.

[123] Head S. A., Shi W., Zhao L. (2015). Antifungal drug itraconazole targets VDAC1 to modulate the AMPK/mTOR signaling axis in endothelial cells. *Proceedings of the National Academy of Sciences*.

[124] Xu F., Cui W. Q., Wei Y. (2018). Astragaloside IV inhibits lung cancer progression and metastasis by modulating macrophage polarization through AMPK signaling. *Journal of Experimental & Clinical Cancer Research*.

[125] Martin M. J., Hayward R., Viros A., Marais R. (2012). Metformin accelerates the growth of BRAF V600E-driven melanoma by upregulating VEGF-A. *Cancer Discovery*.

[126] Marais F. V. N., Valanciute A., Houde V. P. (2012). Aspirin inhibits mTOR signaling, activates AMP-activated protein kinase, and induces autophagy in colorectal cancer cells. *Gastroenterology*.

[127] Salminen A., Kaarniranta K., Kauppinen A. (2016). AMPK and HIF signaling pathways regulate both longevity and cancer growth: the good news and the bad news about survival mechanisms. *Biogerontology*.

[128] Pakravan K., Babashah S., Sadeghizadeh M. (2017). MicroRNA-100 shuttled by mesenchymal stem cell-derived exosomes suppresses in vitro angiogenesis through modulating the mTOR/HIF-1*α*/VEGF signaling axis in breast cancer cells. *Cellular Oncology*.

[129] Zhao L., Fan C., Zhang Y. (2016). Adiponectin enhances bone marrow mesenchymal stem cell resistance to flow shear stress through AMP-activated protein kinase signaling. *Scientific Reports*.

[130] Yang Y., Fan C., Deng C. (2016). Melatonin reverses flow shear stress-induced injury in bone marrow mesenchymal stem cells via activation of AMP-activated protein kinase signaling. *Journal of Pineal Research*.

[131] Seiler C., Stoller M., Pitt B., Meier P. (2013). The human coronary collateral circulation: development and clinical importance. *European Heart Journal*.

[132] Meier D. L., Hu X., Cong L. (2003). Physiological role of AMP-activated protein kinase in the heart: graded activation during exercise. *American Journal of Physiology-Endocrinology and Metabolism*.

[133] Taylor C. W., Ingham S. A., Hunt J. E. A., Martin N. R. W., Pringle J. S. M., Ferguson R. A. (2016). Exercise duration-matched interval and continuous sprint cycling induce similar increases in AMPK phosphorylation, PGC-1*α* and VEGF mRNA expression in trained individuals. *European Journal of Applied Physiology*.

